# The Moderating Role of the Hostile-World Scenario in the Connections Between COVID-19 Worries, Loneliness, and Anxiety

**DOI:** 10.3389/fpsyg.2021.645655

**Published:** 2021-03-16

**Authors:** Yoav S. Bergman, Amit Shrira, Yuval Palgi, Dov Shmotkin

**Affiliations:** ^1^Faculty of Social Work, Ashkelon Academic College, Ashkelon, Israel; ^2^Interdisciplinary Department of Social Sciences, Bar-Ilan University, Ramat Gan, Israel; ^3^Department of Gerontology, University of Haifa, Haifa, Israel; ^4^School of Psychological Sciences and the Herczeg Institute on Aging, Tel Aviv University, Tel Aviv, Israel

**Keywords:** anxiety, COVID-19, hostile-world scenario, loneliness, well-being, worries

## Abstract

The COVID-19 pandemic has had pronounced effects on individuals' psychological well-being around the world. Concerns regarding the consequences of infection, as well as the general uncertainty and governmental regulations have resulted in increased psychological distress among many populations and cultures. In this regard, research has shown that the manner by which individuals perceive such large-scale threats and appraise them significantly contributes to the psychological consequences of such events. According to the Hostile-World Scenario (HWS) model, negative engagement (NE) with such threats weakens one's competence and coping abilities, whereas positive engagement (PE) facilitates resilience and enhances psychological adjustment. Accordingly, the current study examines the moderating role of both NE and PE in the connections of two main features of the current pandemic, COVID-19-related worries and loneliness, with anxiety. Data were collected between March 16 and April 14, 2020, from 1,112 Israelis (age range 17–92, *M* = 46.90, *SD* = 16.46), who provided information regarding COVID-19 health worries, loneliness, and anxiety. A special measure assembled items pertinent to the HWS-NE and HWS-PE throughout the survey. Results demonstrated that both HWS-NE and HWS-PE were significant moderators. COVID-19-related health worries/loneliness were linked with anxiety only among individuals with high HWS-NE, and were non-significant among those with low HWS-NE. Moreover, the positive association between loneliness and anxiety was significantly mitigated by high HWS-PE. The discussion highlights the importance of the HWS for understanding the psychological consequences of COVID-19 and offers practical suggestions, which may aid mental health practitioners in providing assistance and support to the general population.

## Introduction

Since it was first reported in December of 2019, the SARS-CoV-2 virus and the ensuing coronavirus disease (COVID-19), has rapidly spread throughout the world, affecting millions of people, and as of December, 2020, causing 1.5 million deaths (World Health Organization, 2020). While the physical health outcomes of COVID-19 are pronounced and pose a serious health threat, the psychological aspects of the current pandemic cannot be underestimated. Around the world, governments have implemented various forms of preventative health measures, ranging from hand-washing and mandatory masks in public areas to social distancing and lockdown, which greatly disrupt individuals' daily life and take a significant toll on their psychological well-being (e.g., Benke et al., 2020; Ozamiz-Etxebarria et al., 2020).

As will be shown, both loneliness, which may result from social distancing, and the fears and worries concerning the current situation, are linked with increased anxiety symptoms and psychological distress. However, there is relatively little knowledge regarding the individual's personal appraisals and personal resources, which may assist in preserving and maintaining psychological well-being and a sense of meaning and purpose, despite the surrounding threats and adversities. Hence, it is imperative that we deepen our understanding of the intra-psychic mechanisms which underlie the individual's ability to cope with the uncertainties concerning COVID-19.

The current work examines the potential role of the Hostile-World Scenario (HWS; Shmotkin, 2005), which serves as an appraisal system through which the individual assesses negative life conditions, and can serve as a personal resource which may either mitigate the negative consequences of COVID-19-related concerns and loneliness, or alternatively, may exacerbate individuals' psychological distress. Accordingly, the aim of the present research is to examine the moderating role of HWS on the connection of both COVID-19 worries and loneliness with anxiety symptoms during the current pandemic.

A prominent feature of anxiety symptoms, which also constitutes one of the diagnostic criteria for general anxiety disorder (American Psychiatric Association, 2013), is excessive and uncontrollable worries and apprehensions. According to Diefenbach et al. (2001), the phenomenon of worrying is a central element in the experience of anxiety, and cognitions which focus on future threat and danger are seen as precipitating anxious affect (Beck and Emery, 1985). In this regard, research has demonstrated that individuals report high levels of worries during global health crises, such as the 2015 Middle East Respiratory Syndrome pandemic in South Korea (Ro et al., 2017), and this phenomenon was reported even when such outbreaks constituted a relatively remote threat, as could be observed in the reaction of the American population to the Ebola pandemic, which mainly occurred in Africa (see Thompson et al., 2017).

While the content domains of worry in anxiety vary, studies have reported that worries about one's health and possible illnesses constitute a significant venue of concern (Roemer et al., 1997; Diefenbach et al., 2001). While health-related concerns are typically more prominent among older populations, they are nevertheless part and parcel of the phenomenon of anxiety (Becker et al., 2003). In the context of COVID-19, research has demonstrated that concerns regarding COVID-19 are associated with increased anxiety and depression (Barzilay et al., 2020), and in a study which focused specifically on health-related concerns, a positive connection between COVID-19 health concerns and anxiety symptoms was reported among older adults (Bergman et al., 2020).

As previously mentioned, governments around the globe have adopted various public health measures in order to control the outbreak of COVID-19. Many countries have enforced restrictions such as curfews and lockdowns, and strongly promote social distancing in an attempt to manage the public's exposure to the virus. While such measures are understandable from an epidemiological perspective, the psychological ramifications of such social isolation may be linked with increased feelings of loneliness. While loneliness, or the discrepancy between desired and perceived social relationships (see Jeste et al., 2020), has been linked with various negative physical and mental consequences (e.g., Heinrich and Gullone, 2006), it seems that such feelings may play an increasingly significant factor during the COVID-19 pandemic. In this regard, research has demonstrated that higher levels of restriction due to public health measures were associated with increased loneliness (Benke et al., 2020), and that loneliness during the pandemic was a main risk factor for depression, anxiety, and their comorbidity (Palgi et al., 2020). Moreover, Probst et al. (2020) reported that even after lockdown was lifted, stress and loneliness continued to predict depression.

It is important to note that the negative effects of worries and loneliness on the individual's general mental well-being and anxiety levels are not a foregone conclusion. Rather, there are several personal and psychological resources which may contribute to the individual's resilience and sense of competence, thereby mitigating the psychological outcomes of both excessive concerns and feelings of seclusion and isolation. For example, social support has been suggested as an important factor for reducing loneliness during COVID-19 (Saltzman et al., 2020), and research has demonstrated that this factor is indeed associated with lower levels of both loneliness (Grey et al., 2020) and anxiety symptoms (Ao et al., 2020). Moreover, personal self-esteem was also found to be linked with reduced COVID-19 worries, loneliness, and anxiety (Rossi et al., 2020). Additionally, Dawson and Golijani-Moghaddam (2020) found that during COVID-19, individuals who adopted an avoidant coping style (characterized by engaging in self-distraction, denial, or behavioral disengagement) reported reduced psychological well-being and increased anxiety and COVID-19 worries, whereas adopting an approach coping style (i.e., engaging in active coping, usage of instrumental/emotional support, positive reframing, and planning) was associated with increased well-being and reduced anxiety and worries. More importantly, however, they also demonstrated that psychological flexibility, or one's personal ability to recognize and adapt to the ever-changing situational demands during the pandemic, was associated with reduced COVID-19 worries and anxiety.

The aforementioned findings seem to indicate that individuals can rely on various psychological mechanisms in order to deal with the threats posed by COVID-19. Furthermore, the COVID-19 pandemic, representing a scenario of dangerous inflictions and eventualities, challenges the individual's psychological forces to sustain resilience and regain well-being (Wong, 2020). Thus, the utility of resilient mechanisms may depend on the manner by which individuals perceive actual and/or potential threats, and the extent to which such threats compromise their sense of well-being and meaning in life. Accordingly, we examine the HWS model (Shmotkin, 2005) and explore its possible role in counteracting adverse psychological effects of worries and loneliness during the COVID-19 pandemic.

The HWS is a core construct in a larger conceptual framework addressing the pursuit of happiness in a hostile world (see also Shmotkin and Shrira, 2012, 2013). According to this conception, the imperative of maintaining well-being and meaning in life is constantly challenged by the dangers of living in a potentially hostile world. In order to manage this existential, ever-present psychological tension, individuals formulate their own personal HWS, which is one's image of actual or potential threats to one's life or, more broadly, to one's physical and mental integrity. The HWS aggregates self-beliefs about critical threats in life, such as natural or human-made disasters, accidents, serious illnesses, economic deprivation, suffering and separation of loved ones, and death. The HWS constitutes a personal appraisal system which monitors potential negative conditions, or, if such a condition is present, scans for even worse possible events or circumstances. When the HWS is activated in an adaptive manner, it assists the individual to remain vigilant and alert in his/her striving to remain safe. Thus, the HWS proved capable of detecting changes in physical and mental health occurring in the past recent years (Lifshitz et al., 2020) as well as predicting changes in similar health outcomes occurring in subsequent years (Shmotkin et al., 2016). However, individuals with extremely activated HWS may operate in a constant state of survival within a perceivably disastrous world.

According to Shmotkin et al. (Shmotkin et al., 2016; see also Shrira et al., 2011; Shrira, 2015), the HWS offers individuals two main perspectives of experiencing and dealing with life adversities. On the one hand, the individual may adopt a strategy of *negative engagement* (HWS-NE) with his/her representations of the hostile world. This type of engagement entails the weakening of one's sense of competence, thus rendering individuals vulnerable to worries, fears, pessimism, and hopelessness. On the other hand, a *positive engagement* (HWS-PE) with one's representations of the hostile world involves strengthening one's sense of competence, thus leading individuals to adopt proactive behaviors and optimism. In this regard, research has demonstrated that HWS-NE was associated with reduced subjective well-being and sense that life bears meaning, whereas HWS-PE was linked with increased well-being and meaning in life (Shrira et al., 2011). In this vein, upon examining the prospect of an Iranian nuclear attack on Israel, Shrira (2015) reported that HWS-NE was associated with an increased sense of threat from such an attack, whereas HWS-PE demonstrated the opposite pattern.

It seems, therefore, that the HWS offers a theoretical paradigm, which may elucidate how individuals, who experience worries concerning COVID-19 or loneliness due to reduced social contact, cope with threats of the hostile world at large. We surmise that individuals who view potential threats as debilitating or incapacitating (i.e., individuals with high levels of HWS-NE) would display higher levels of subsequent anxiety, whereas those who are able to face such threats more positively and proactively (i.e., individuals with high levels of HWS-PE) would demonstrate reduced anxiety. Accordingly, the following hypotheses were formulated:

(H1): A main effect of COVID-19 worries will be discovered, as high levels of worries will be associated with high levels of anxiety.
(H2): A main effect of loneliness will be found, as high levels of loneliness will be associated with high levels of anxiety.
(H3): High levels of HWS-NE will be associated with increased anxiety levels, whereas high levels of HWS-PE will be associated with reduced levels of anxiety.
(H4): HWS will moderate the connections between COVID-19 worries, loneliness, and anxiety. More specifically, the respective links of worries and loneliness with anxiety are hypothesized to be more pronounced among individuals with high levels of HWS-NE, and less pronounced among individuals with high levels of HWS-PE.

## Materials and Methods

### Participants and Procedure

Data were collected in Israel between March 16 to April 14, 2020, using the Qualtrics web-based public platform in the midst of the first lock-down period of COVID-19 (which began on March 14, and was lifted, to a certain extent, on April 19). On the last day of data collection, 12,361 Israelis were tested positive for COVID-19 and 123 had died. The sample included 1,112 people between the ages of 17 and 92 (mean age = 46.90, *SD* = 16.46). Most of them were women (*n* = 838, 75.4%), married or cohabitating (*n* = 799, 71.9%), and most had an academic degree (*n* = 841, 75.8%). Of the participants, 201 (18.3%) reported to have been diagnosed with chronic medical condition known to be related to increased risk of death due to COVID-19 complications. The majority rated their health (*n* = 875, 78.7%) and economic status (*n* = 654, 59.0%) as good or very good.

The online questionnaire was disseminated across multiple social media resources and contact lists provided by organizations. All participants provided an informed consent. Ethical approval was received from the Institutional Review Board at the third author's University.

### Measures

Participants completed background characteristics, including age, gender, marital status, and education (rated from 1 = without formal education to 6 = formal tertiary education). They noted whether they have been diagnosed with chronic medical conditions known to be related to increased risk of death due to COVID-19 complications (i.e., cardiovascular disease, diabetes, chronic respiratory disease, hypertension, and cancer), and rated their health and economic status on scales ranging from 1 (*not good at all*) to 5 (*very good*). Participants further reported their level of exposure to six COVID-19 pandemic related events [i.e., being tested positive for the coronavirus, being (or having been) in self-isolation, knowing family members, friends, or other people who were tested positive, knowing people in self-isolation; exposure score was the sum of events]. Participants further reported whether they have changed 11 behaviors due to the COVID-19 pandemic (i.e., avoiding shaking hands, avoiding hugs, keeping physical distance from others, avoiding social events, going out less frequently, avoiding inviting or meeting with people, using a mask or gloves, avoiding going to public places, washing hands more often, buying more food and water than usual, canceling/changing significant plans; behavioral change score was the sum of behaviors changed).

Participants completed the additional below measures while being asked to relate to their feelings and symptoms during the COVID-19 pandemic and due to it.

COVID-19 related health worries were assessed by four items which assess concerns regarding possible COVID-19 infection of one's self/close people/relatives, or infecting others in case of having COVID-19 (Bergman et al., 2020). Items were rated on a 5-point scale ranging from 1 (*not at all*) to 5 (*almost always*). A mean score was calculated, and higher scores reflected increased worries. Internal reliability was good (α = 0.84).

Loneliness was assessed with the 3-item version of the UCLA Loneliness Scale (Hughes et al., 2004). Items (e.g., *how often do you feel that you lack companionship?*) were rated on a 5-point scale ranging from 1 (*not at all*) to 5 (*almost always*). The loneliness score was the mean of ratings, and higher scores reflected higher loneliness. The Hebrew version of the scale was used in previous studies (Spitzer et al., 2019). Internal reliability was good (α = 0.86).

Anxiety symptoms were assessed with the 7-item Generalized Anxiety Disorder (GAD-7) scale (Spitzer et al., 2006). Participants rated their symptoms (e.g., *feeling nervous, anxious, or on edge*) during the last 2 weeks on a 4-point scale (0 = *not at all* to 3 = *almost every day*). Ratings were summed with higher scores reflecting increased anxiety. The Hebrew version of the scale was used in previous studies (Shrira et al., 2019). Internal reliability was good (α = 0.93).

The current HWS measure was specifically derived from the current dataset (for other HWS operationalizations, see Shrira et al., 2011; Shenkman and Shmotkin, 2013; Shrira, 2015; Shmotkin et al., 2016; Lifshitz et al., 2020). It included eight items that corresponded to the conceptualization of HWS-NE, namely, the weakening of the individual's competence because of the encounter with the HWS representations. Four additional items corresponded to the conceptualization of HWS-PE, namely, the maintenance or strengthening of the individual's competence because of the encounter with the HWS representations. These items (see Table 1) were scattered in various sections of the survey. Selection of items into the HWS measure was made by consensus of two researchers acquainted with the HWS concept. The original 5-point rating scales was similar among HWS items. Therefore, HWS-NE and HWS-PE scores were the mean of ratings in their respective items, and higher scores reflected increased HWS-NE or HWS-PE. Notably, the measures to which the 12 items originally belonged were not included in the current work.

**Table 1:** Factor analysis of HWS items.

	**Factor loadings**	
**Items**	**1**	**2**
	**Negative engagement**	**Positive engagement**
1. My medical problems make me very anxious	**0.52**	−0.02
2. My financial situation makes me very anxious	**0.49**	0.07
3. I have a feeling my life is coming to an end^a^	**0.31**	−0.10
4. I think a lot about death^b^	**0.62**	−0.01
5. I am very afraid of death^b^	**0.45**	−0.12
6. I feel afraid for my safety^c^	**0.75**	0.03
7. I feel worried about the safety of others^c^	**0.54**	0.13
8. I feel like I am about to lose control of my emotions^c^	**0.46**	−0.21
9. I tend to bounce back after illness, injury or other hardships^d^	−0.08	**0.65**
10. I am not easily discouraged by failure^d^	0.005	**0.44**
11. I think of myself as a strong person when dealing with life's challenges and difficulties^d^	0.06	**0.88**
12. I am able to handle unpleasant or painful feelings like sadness, fear, and anger^d^	−0.03	**0.75**
Eigen-value	3.01	1.39
Variance explained (after rotation)	25.13	11.60

*Presented are factor loadings obtained in a principal component analysis using varimax rotation constrained to two factors. After a listwise deletion of missing values, the number of cases in the analysis was 939. HWS, hostile-world scenario*.

a *This single item is based on the item suggested by Kotter-Grühn et al. (2010)*.

b *Items were taken from the Fear of Death scale (Carmel and Mutran, 1997)*.

c *Items were taken from the Peritraumatic Distress Inventory (PDI; Brunet et al., 2001)*.

d *Items were taken from the Connor–Davidson Resilience Scale (CD-RISC; Campbell-Sills and Stein, 2007). All other items were specifically generated for the current survey*.

An exploratory factor analysis (a principal component extraction with a varimax rotation) of the 12 HWS items provided internal validity information about the thematic composition of the current HWS measure (see Table 1). A solution constrained to two factors appeared most feasible according to the following criteria: (1) the factors' eigen-values were >1; (2) items within each factor had loadings of at least 0.30 with a difference of at least 0.25 from their loadings on other factors. Eight items, labeled *HSW-NE*, loaded on Factor 1. These items related to being restricted by powerful life conditions (e.g., loss of health or financial security), by the fear of death, or by otherwise perceived lack of control. Four other items, labeled *HSW-PE*, loaded on Factor 2. These items related to feelings that one is capable of overcoming hardships or even feels strengthened by them. Cronbach's α was 0.76 and 0.77 for HWS-NE and HWS-PE, respectively.

### Data Analysis

First, we calculated means and standard deviations, frequencies and percentages as basic descriptive statistics. Pearson and point-biserial correlation coefficients were used to describe the relationships between these variables. Second, four complementary hierarchical regressions were conducted for testing the study hypotheses. In each of the analyses, anxiety symptoms were regressed on demographic variables (age, gender, education, marital status, and subjective economic status) in Step 1, self-rated health, medical conditions related to increased risk of death due to COVID-19 complications, COVID-19 related exposure, and behavioral change during the pandemic in Step 2, HWS-NE or HWS-PE in Step 3, and either COVID-19 related health worries or loneliness in Step 4. The last step included the interaction between the variables in Steps 3 and 4. The interactions were probed using Model 1 of the PROCESS 3.4 computation plugin (Hayes, 2018) in two manners. First, we examined the connections between the predictors (COVID-19 related health worries/loneliness) and anxiety at low/high levels of the moderator (HWS-NE/HWS-PE). Second, in order to provide a more detailed account of the aforementioned links, we employed the Johnson-Neyman technique, which provides confidence intervals for the connection between the predictors and anxiety at various values of the moderator.

## Results

Relatively few participants reported being tested positive for COVID-19 or being in self-isolation (*n* = 136, 13.3%), yet most knew someone who tested positive or was in self-isolation (*n* = 808, 75.6%). Almost all respondents (99.4%) reported changing at least one behavior due to the pandemic ranging from using mask or gloves (the use of masks was non-compulsory in Israel until April 12th, *n* = 398, 35.8%) to washing hands more often (*n* = 987, 88.8%).

Mean scores for the study variables as well as correlations can be found in Table 2. High levels of HWS-NE were associated with increased COVID-19 related health worries, loneliness, and anxiety symptoms (0.31 ≤ *r* ≤ 0.63, *p*s < 0.001), whereas high levels of HWS-PE were related to reduced worries, loneliness, and anxiety (−0.26 ≤ *r* ≤ −0.14, *p*s < 0.001). Older respondents, men, those with higher education, better self-rated economic and health status reported less HWS-NE and more HWS-PE. Behavioral change during the pandemic was related to both high HWS-NE and high HWS-PE. Finally, HWS-NE and HWS-PE were moderately and negatively correlated.

**Table 2:** Descriptive statistics and correlations among study variables.

		***M*/%**	***SD***	**1**	**2**	**3**	**4**	**5**
1.	HWS-NE	2.18	0.63	–				
2.	HWS-PE	3.94	0.71	−0.30^***^	–			
3.	COVID-19 related health worries	3.25	0.94	0.47^***^	−0.14^***^	–		
4.	Loneliness	2.36	1.00	0.31^***^	−0.19^***^	0.12^***^	–	
5.	Anxiety symptoms	5.16	5.40	0.63^***^	−0.26^***^	0.40^***^	0.29^***^	–
6.	Age	46.9	16.46	−0.19^***^	0.10^**^	−0.23^***^	−0.14^***^	−0.26^***^
7.	Gender^a^	75.4	–	0.12^***^	−0.09^**^	0.09^**^	0.08^*^	0.14^***^
8.	Education	5.58	0.82	−0.10^**^	0.07^*^	−0.08^*^	−0.07^*^	−0.02
9.	Marital status^b^	71.9	–	−0.06	0.06	−0.04	−0.16^***^	−0.01
10.	Subjective economic status	3.64	0.89	−0.28^***^	0.12^***^	−0.09^**^	−0.12^***^	−0.16^***^
11	Self-rated health	4.14	0.91	−0.23^***^	0.14^***^	−0.05	−0.06	−0.07^*^
12.	Medical conditions^c^	18.3	–	0.02	0.02	−0.07^*^	−0.05	−0.08^*^
13.	Exposure to COVID-19	1.36	1.03	0.01	0.03	0.09^**^	0.07^*^	0.02
14.	Behavioral change in COVID-19	8.17	2.31	0.16^***^	0.09^**^	0.24^***^	0.12^***^	0.14^***^

*N ranged 915–1,112. Correlation values are Pearson coefficients, except for values involving items 7, 9, and 12, which are point-biserial coefficients. a, female; b, married or cohabitating; c, diagnosed with chronic medical condition known to be related to increased risk of death due to COVID-19 complications. HWS-NE, Hostile-world scenario-negative engagement; HWS-PE, Hostile-world scenario-positive engagement*.

* p < 0.05;

** p < 0.01;

*** *p < 0.001*.

Table 3 presents the results of the main regression analyses predicting anxiety by HWS and COVID-19 related health worries. Across regressions, being younger, female, married, and having lower economic status were linked with higher anxiety level. After controlling for the effect of Step 1 variables, lower self-rated health and more behavioral change during the pandemic were related to higher anxiety. In line with the first hypothesis, high levels of COVID-19 worries (see Table 3 for regression coefficients) were associated with increased anxiety symptoms. Moreover, in accordance with the third hypothesis, HWS-NE was associated with high levels of anxiety, whereas the opposite direction was found for HWS-PE. Finally, in partial corroboration of the fourth hypothesis, the interaction between HWS-NE and COVID-19 related health worries was significant. The interaction between HWS-PE and COVID-19 related health worries was marginally significant.

**Table 3:** Summary of results obtained in regression analyses for predicting anxiety by HWS and COVID-19 health worries.

	**Anxiety**					
	**HWS-NE**			**HWS-PE**		
	**Δ*R*^**2**^**	***B***	**β**	**Δ*R*^**2**^**	***B***	**β**
Step 1	0.09^***^			0.09^***^		
Age		−0.08	−0.23^***^		−0.08	−0.23^***^
Gender^a^		1.56	0.13^***^		1.62	0.13^***^
Education		0.17	0.03		0.15	0.02
Marital status^b^		0.95	0.08^*^		0.86	0.07^*^
Subjective economic status		−0.69	−0.11^**^		−0.70	−0.12^***^
Step 2	0.04^***^			0.04^***^		
Self-rated health		−0.81	−0.14^***^		−0.78	−0.13^***^
Medical conditions^c^		−0.65	−0.05		−0.61	−0.04
Exposure to COVID-19 events		−0.12	−0.02		−0.06	−0.01
Behavioral change in COVID-19		0.43	0.17^***^		0.42	0.17^***^
Step 3	0.31^***^			0.05^***^		
HWS-NE/PE		5.20	0.62^***^		−1.71	−0.23^***^
Step 4	0.01^**^			0.07^***^		
COVID-19 related health worries		0.56	0.10^**^		1.67	0.29^***^
Step 5	0.01^***^			0.003^+^		
HWS-NE/PE × COVID-19 related health worries		0.56	0.12^***^		−0.27	−0.05^+^

*N ranged 893–916. Only additional variables are shown in the results of Steps 2–5. a, women; b, married or cohabitating; c, diagnosed with chronic medical condition known to be related to increased risk of death due to COVID-19 complications. HWS-NE, Hostile-world scenario-negative engagement; HWS-PE, Hostile-world scenario-positive engagement*.

+ p < 0.10;

* p < 0.05;

** p < 0.01;

*** *p < 0.001*.

**Figure 1:**
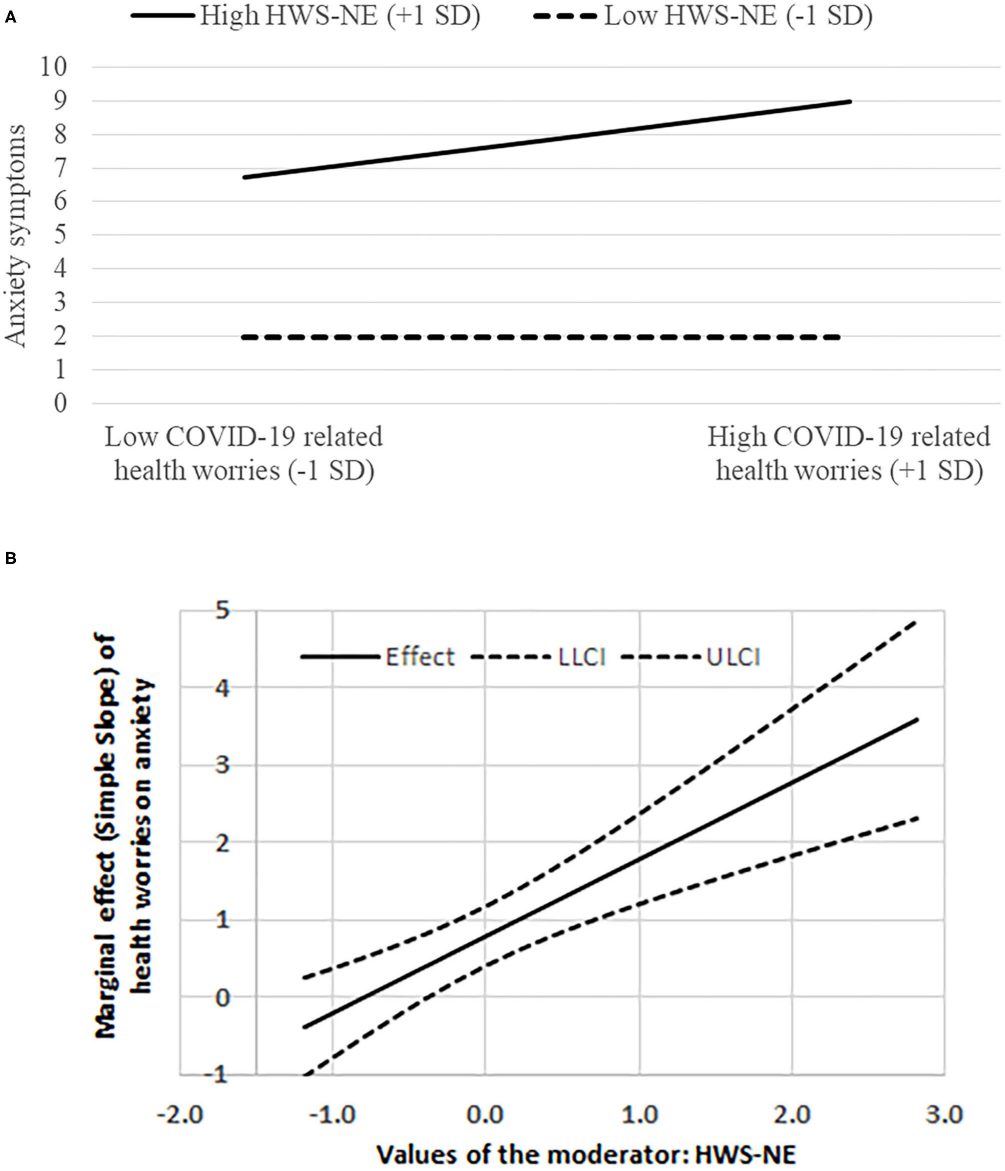
**(A)** The interaction between HWS-NE (hostile-world scenario-negative engagement) and COVID-19 related health worries on anxiety symptoms. **(B)** The marginal effect (simple slope) of COVID-19 related health worries on anxiety symptoms at various levels of HWS-NE; LLCI/ULCI = Lower/upper 95% limit for confidence interval.

Upon examining the significant interaction using PROCESS (Hayes, 2018, Model 1), we discovered that when levels of HWS-NE were at −1 SD below the mean (i.e., low HWS-NE), the relationship between COVID-19 related health worries and anxiety was non-significant [*B* = −0.001, *t*_(880)_ = −0.004, *p* = 0.99]. However, for those with high levels of HWS-NE (+1 SD above the average) the COVID-19 related health worries-anxiety association was significant [*B* = 1.20, *t*_(880)_ = 5.66, *p* < 0.0001; see Figure 1A]. Using the Johnson-Neyman technique, we found that when levels of HWS-NE were slightly below the mean and lower, the marginal effect (simple slope) of COVID-19 related health worries on anxiety was insignificant. However, this effect turned significant and positive, and increased upon higher levels of HWS-NE [see Figure 1B for simple slopes and 95% confidence interval resulted from the bootstrapping resampling technique (*n* repeats = 2,000)].

Table 4 presents the results of the main regression analyses predicting anxiety by HWS and loneliness. After controlling for the effect of Steps 1–3 variables, loneliness was related to higher anxiety, which is in line with the second hypothesis. Moreover, the interaction between HWS-NE and loneliness was significant, as was the interaction between HWS-PE and loneliness.

**Table 4:** Summary of results obtained in regression analyses for predicting anxiety by hws and loneliness.

	**Anxiety**					
	**HWS-NE**			**HWS-PE**		
	**Δ*R*^**2**^**	***B***	**β**	**Δ*R*^**2**^**	***B***	**β**
Step 1	0.09^***^			0.10^***^		
Age		−0.08	−0.24^***^		−0.08	−0.25^***^
Gender^a^		1.34	0.11^***^		1.36	0.11^***^
Education		0.25	0.04		0.24	0.07
Marital status^b^		0.86	0.07^*^		0.80	0.07^*^
Subjective economic status		−0.80	−0.13^***^		−0.79	−0.13^***^
Step 2	0.04^***^			0.04^***^		
Self-rated health		−0.84	−0.14^***^		−0.81	−0.14^***^
Medical conditions^c^		−0.46	−0.03		−0.42	−0.03
Exposure to COVID-19 events		−0.05	−0.01		−0.03	−0.01
Behavioral change in COVID-19		0.38	0.16^***^		0.36	0.16^***^
Step 3	0.31^***^			0.04^***^		
HWS-NE/PE		5.26	0.62^***^		−1.59	−0.21^***^
Step 4	0.01^***^			0.04^***^		
Loneliness		0.51	0.09^***^		1.12	0.21^***^
Step 5	0.01^**^			0.01^*^		
HWS-NE/PE × Loneliness		0.34	0.08^**^		−0.39	−0.08^**^

*N ranged 1,041–1,063. Only additional variables are shown in the results of Steps 2–5. a, women; b, married or cohabitating; c, diagnosed with chronic medical condition known to be related to increased risk of death due to COVID-19 complications. HWS-NE, Hostile-world scenario-negative engagement; HWS-PE, Hostile-world scenario-positive engagement*.

* p < 0.05;

** p < 0.01;

*** *p < 0.001*.

**Figure 2:**
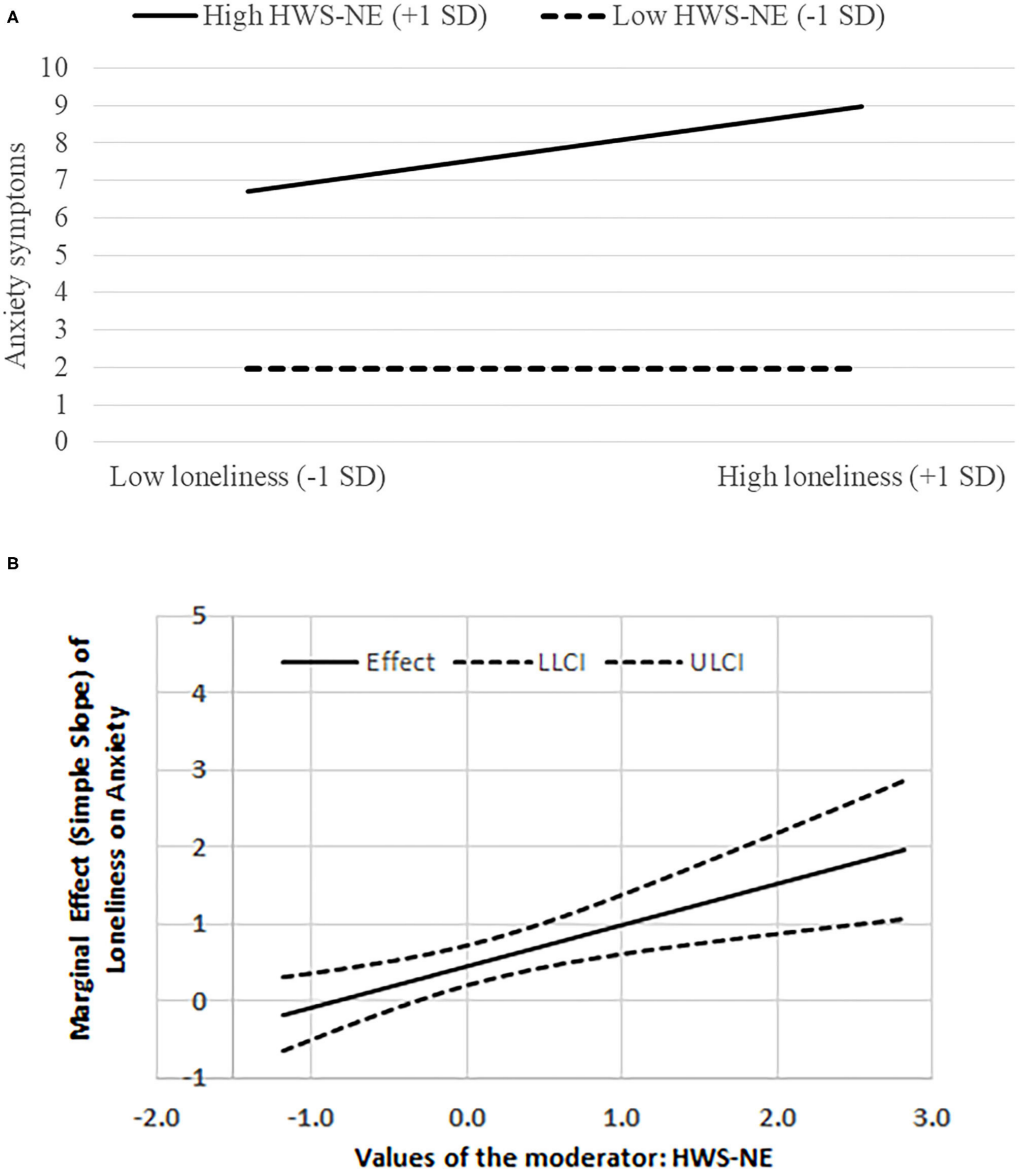
**(A)** The interaction between HWS-NE (hostile-world scenario-negative engagement) and loneliness on anxiety symptoms. **(B)** The marginal effect (simple slope) of loneliness on anxiety symptoms at various levels of HWS-NE; LLCI/ULCI = Lower/upper 95% limit for confidence interval.

**Figure 3:**
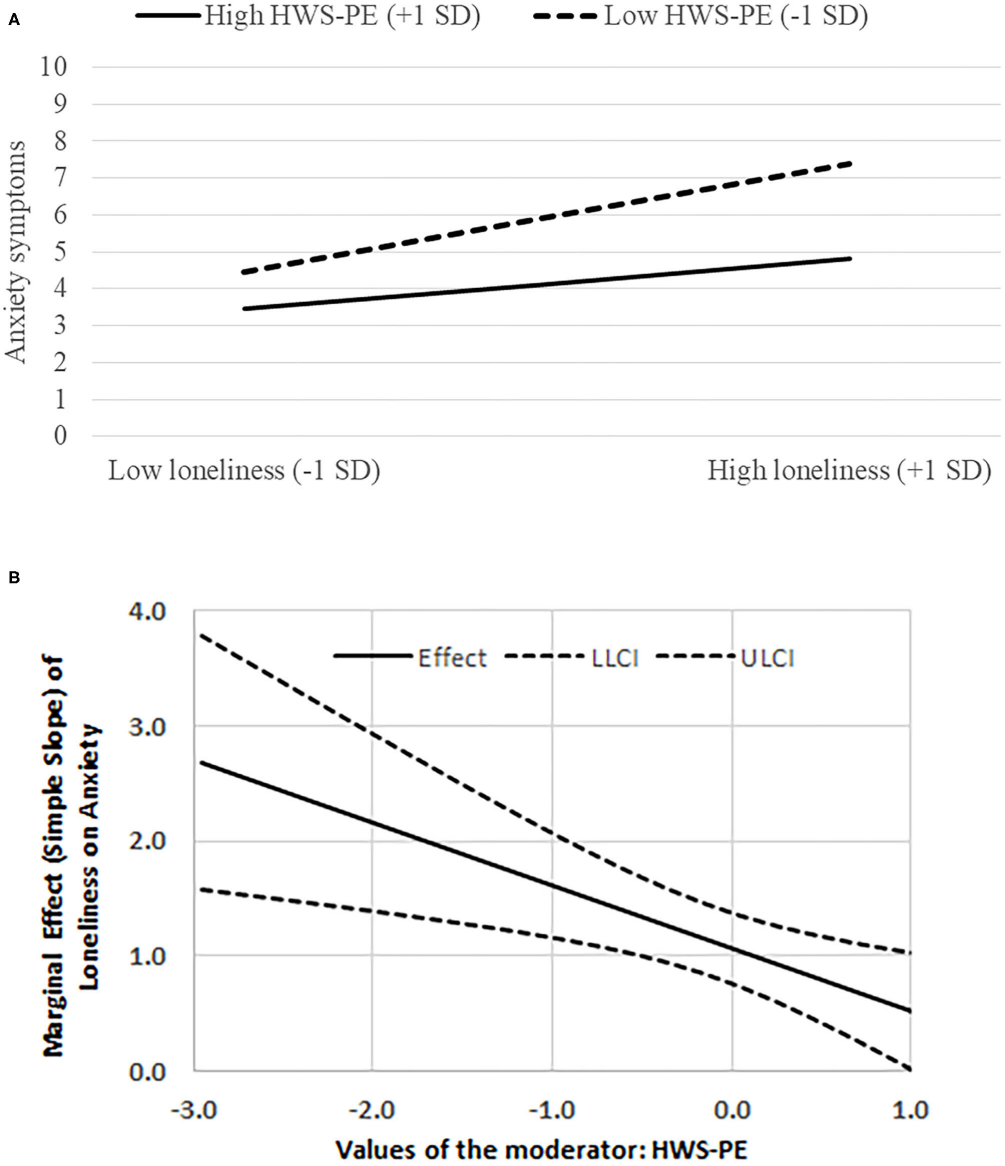
**(A)** The interaction between HWS-PE (hostile-world scenario-positive engagement) and loneliness on anxiety symptoms. **(B)** The marginal effect (simple slope) of loneliness on anxiety symptoms at various levels of HWS-PE; LLCI/ULCI = Lower/upper 95% limit for confidence interval.

Upon probing the interactions, we found that among individuals with low HWS-NE, the relationship between loneliness and anxiety was non-significant [*B* = 0.12, *t*_(1028)_ = 0.68, *p* = 0.50]. However, for those with high levels of HWS-NE the loneliness-anxiety association was significant [*B* = 0.79, *t*_(1028)_ = 4.95, *p* < 0.0001; see Figure 2A]. Moreover, when HWS-PE was high, the relationship between loneliness and anxiety was significant [*B* = 0.68, *t*_(1050)_ = 3.16, *p* = 0.001]. However, for those with low levels of HWS-PE the loneliness-anxiety association was much stronger [*B* = 1.46, *t*_(1050)_ = 7.49, *p* < 0.0001; see Figure 3A]. Upon employing the Johnson-Neyman method, these trends were corroborated; First, the loneliness-anxiety connection was not significant among individuals with very low levels of HWS-NE. However, when the moderator value increased, the loneliness-anxiety association was positive and significant, and grew more pronounced as HWS-NE levels increased (see Figure 2B). The expected opposite effect was found with respect to varying values of HWS-PE. A positive significant loneliness-anxiety association, yet decreasing, was found as long as HSW-PE values were low to high and became insignificant only when HSW-PE was at its highest observed value (see Figure 3B). It should be noted that our alternative decomposition of the interaction effect, which was based on an expected curvilinear association, resulted in similar associations with anxiety.

## Discussion

The COVID-19 has rapidly become a global health pandemic, and notwithstanding the physical and health-related ramifications of the disease, the psychological consequences of the current crisis are dire and pronounced. As people have had to significantly change their lives, due to both personal health-related decisions and governmental regulations, the sense of well-being, and perhaps the basic security of being able to conduct a relatively normal life, are greatly compromised. Facing this existential challenge of disruptive threats, individuals may respond in varying ways, and there is a need to further our understanding with regard to the psychological mechanisms, which underlie the manner by which one perceives and reacts to the threats posed by COVID-19.

The aim of the present study was to examine the links between COVID-19-related worries, loneliness, and anxiety. By adopting the concept of the HWS (Shmotkin, 2005), we aimed to explore whether the individual's coping modes with imminent adversities moderates the effects of current worries and loneliness on anxiety symptoms. As noted in the introduction, the conceptualization of the HWS refers to an aggregate of self-beliefs that formulate the image that each individual has about actual or potential threats to one's life or, more broadly, to one's physical and mental integrity (Shmotkin, 2005, 2011; Shmotkin and Shrira, 2012, 2013). In the HWS measure used in the current study, these threats are represented by themes of health problems and illness, death, financial problems, safety issues, painful feelings, failure and other disabilities and life difficulties. The concept of the HWS is a dynamic construct whereby individuals may encounter HWS representations by either negative engagement (HWS-NE), marked by the sense of losing competence in the face of life adversities, or by positive engagement (HWS-PE), marked by the sense of gaining competence in the face of life adversities. Hence, both HWS-NE and HWS-PE are adaptive modes for scanning and appraising the potentiality and implications of critical threats for the individual (Shrira et al., 2011; Shmotkin et al., 2016; Lifshitz et al., 2020).

In this regard, it is also important to note that while the items comprising the HWS in the current study may be linked to anxiety (HWS-NE) or resilience (HWS-PE), both HWS-NE and HWS-PE cannot be seen as merely a reflection of one's state of distress or personal inner strengths; Rather, the HWS mainly relates to *existential human concerns*, such as the potentially critical inflictions of vulnerability (illness, pain, loss of personal independence), victimization (to violence, collective disasters) and imminent death (of self and close ones). These existential inflictions may indeed strengthen one's view of the world as a hostile environment, and often involve dialectical forces (thus, HWS-NE and HWS-PE, despite their moderately negative correlation, do not exclude each other). As the imperative of maintaining well-being is constantly challenged by the dangers in a potentially hostile world, the HWS model, in its fuller scope, is closely interwoven into *positive psychology* constructs (subjective well-being, meaning in life) which are postulated to hold reciprocally regulatory relations with the HWS.

In line with the first two hypotheses, high levels of both COVID-19 worries and loneliness were associated with increased anxiety symptoms. These results corroborate previous findings which link both worries (Barzilay et al., 2020; Bergman et al., 2020) and loneliness (Palgi et al., 2020; Probst et al., 2020) with increased psychological distress during the COVID-19 pandemic. However, our findings here may provide important directions for understanding how the public reacts to the ever-changing nature of this global crisis. According to Asmundson and Taylor (2020), concerns and anxieties about one's health may affect how an individual responds to public health strategies used to manage epidemics and pandemics (see also Taylor, 2019). In other words, individuals who demonstrate high health anxiety may view medical facilities as a source of contamination and choose to avoid them altogether, or alternatively, due to their fears and concern, may frequently visit doctors and undergo frequent COVID-19 tests, thereby increasing the burden of health care resources. Similarly, loneliness has been found to be associated with increased usage of health care facilities among older adults (e.g., general practitioner visits; Burns et al., 2020). In light of the limited medical resources at hand during this health crisis, understanding the connections between health worries, loneliness, and anxiety may bear an important contribution not only for alleviating psychological distress, but also for managing how such resources are distributed among the general population.

As previously noted, the levels of anxiety and psychological distress are closely linked with the manner by which individuals perceive adversity as a serious threat which greatly compromises their sense of confidence and self-assurance, or alternatively, as a challenge which they are capable of dealing with. Accordingly, we found that the former perception, as indicated in high levels of HWS-NE, is associated with increased anxiety, whereas the latter, denoted by high levels of HWS-PE, is linked with reduced anxiety.

More importantly, both modes of engagement with the HWS significantly moderated the effects of COVID-19 worries and loneliness on anxiety symptoms. Thus, among individuals who demonstrated low levels of HWS-NE, COVID-19 worries and loneliness were not associated with anxiety symptoms, and these connections remained significant only for individuals reporting high HWS-NE levels. In parallel, while the loneliness-anxiety link was significant for individuals with both high and low levels of HWS-PE, the connection between the variables was considerably more pronounced for those with low HWS-PE levels. It should also be noted that although the interaction of worries with HWS-PE was only marginally significant (*p* = 0.06), the same trend was observed (i.e., that the effect of COVID-19 worries on anxiety was stronger among individuals with low HWS-PE). It should be noted that despite its significant role, it seems that HWS-PE is less powerful, both as a main predictor and as a moderator, than HWS-NE. This is in line with previous findings which indicate that HWS-NE plays a more important role in the context of meaning in life (Shrira et al., 2011), and as a determinant of perceived threat and psychological distress (Shrira, 2015).

These findings highlight the importance of examining how the manner by which individuals perceive difficulties, challenges and adversities, affects the psychological ramifications of COVID-19. It seems that when individuals generally adopt a *negative* engagement with their HWS (i.e., sensing decreased competence vis-à-vis critical dangers and possible death), their approach may exacerbate the current psychological effects of worries and loneliness. This result appears, unfortunately, quite common during the COVID-19 pandemic (Benke et al., 2020; Bergman et al., 2020). On the other hand, when individuals generally adopt a *positive* engagement with their HWS (i.e., sensing increased competence vis-à-vis critical dangers and possible death), they appear to deal more effectively with negative emotions—either in the larger sphere of a hostile and unpredictable world, or in the tangibly adverse conditions of the current pandemic. Notably, while HWS-NE and HWS-PE are negatively correlated, the relatively modest correlation (*r* = −0.30) indicates that the two modes are not direct opposites, and may co-exist in varying degrees in the same individuals (Palgi et al., 2015). Indeed, as the HWS model explicates (Shmotkin et al., 2016), the HWS-NE does have an adaptive value when appropriately activated, mainly in detecting imminent dangers and promoting preparedness for them (in this vein appears the positive correlation between HWS-NE and behavioral change in COVID-19). Altogether, the HWS model, as depicted here, is in line with the Positive Psychology 2.0 conception, which addresses the dialectical interplay between positive and negative dimensions in inducing human adaptation (Wong, 2019).

Our results can be seen in consistence with Folkman and Lazarus' transactional theory of stress and coping (Folkman and Lazarus, 1984). According to this theory, individuals continually appraise their environment, and when a given stimulus is perceived as threatening, challenging, or potentially harmful, the subsequent distress elicits differential coping strategies (see also Biggs et al., 2017). In the current context, there is little doubt that COVID-19 represents such a stimulus which, in the primary appraisal state, prompts a perception of a situation which may cause significant harm to the individual, and thus poses a significant threat. Accordingly, the HWS is employed in the secondary appraisal phase, in which the individual determines how he/she can manage the resulting distress, while relying on personal and situational resources. When individuals demonstrate high levels of HWS-NE, the distress resulting from COVID-19-related health worries and loneliness is exacerbated, whereas when the situation is perceived as manageable, as portrayed by high levels of HWS-PE, the distress is reduced. In other words, an appraisal of a dangerous situation will, when combined with insufficient resources, increase distress. However, when relevant personal resources are available, the subsequent distress, even when the initial appraisal is threatening, will be less pronounced.

Several limitations of the present study should be noted. First, as our results are based on cross-sectional data, causality cannot be implied. While worries are described as an important element of anxiety disorders (American Psychiatric Association, 2013), and research has demonstrated a causal connection between loneliness and anxiety (Domènech-Abella et al., 2019), it is nevertheless important to examine the study model using longitudinal data both during and after the COVID-19 pandemic. Along this line of thought, since COVID-19 is an ongoing pandemic, both worries and loneliness may be subjected to variations as the situation progresses and governmental restrictions are repetitively enforced and lifted, and it is imperative that the described connections should be examined further in the future. Moreover, as our data collection began only 2 days after the first lockdown in Israel, it is possible that anxiety levels may have been affected by COVID-19 factors not directly associated with the lockdown, and this emphasizes the need to re-examine our study model in the future. Second, it is important to take into consideration additional factors which may affect how individuals perceive adversity. As the full HWS model considers subjective well-being and meaning in life as complementary and regulatory systems (Shmotkin and Shrira, 2013), further research should examine expected interactions between these positive systems and the HWS as potential determinants of individuals' variability in responding to the currently prevailing crisis.

Despite its limitations, our study provides a further understanding of how COVID-19-related experiences are linked with anxiety, and how personal perspectives on the hostile world may both reduce and exacerbate the psychological effects of such experiences. This work may thus be instructive to mental health practitioners, who need to comprehend whether the particular psychological consequences of the almost ubiquitous concerns and social isolation experienced during COVID-19 would be incorporated in a more generalized sense of vulnerability or, rather, resilience. Perhaps more importantly, as humans who are often faced with concerns, social isolation, and loneliness, the findings may invite us all to examine, within ourselves, how our judgment of the pandemic shapes our ability to cope with existential challenges in a threatening and unpredictable world.

## Data Availability Statement

The raw data supporting the conclusions of this article will be made available by the authors, without undue reservation.

## Ethics Statement

The studies involving human participants were reviewed and approved by University of Haifa IRB. The patients/participants provided their written informed consent to participate in this study.

## Author Contributions

YB wrote the paper. AS analyzed the data and contributed the methodological parts. YP collected the data. DS oversaw the process and provided valuable theoretical insights. All authors were responsible for the conceptualization of the study model, contributed to the article, and approved the submitted version.

## Conflict of Interest

The authors declare that the research was conducted in the absence of any commercial or financial relationships that could be construed as a potential conflict of interest.
